# Development of a mouse IgA monoclonal antibody-based enzyme-linked immunosorbent sandwich assay for the analyses of RBP4

**DOI:** 10.1038/s41598-018-20762-x

**Published:** 2018-02-07

**Authors:** Nam Seok Lee, Han Soo Kim, Se Eun Park, Matthias Blüher, Cheol-Young Park, Byung-Soo Youn

**Affiliations:** 1 Green Cross Medical Science, Umseong-gun, Chungcheongbuk-do South Korea; 2 Department of Biomedical Sciences, Catholic Kwandong University College of Medicine, Gangneung-si, Gangwon-do South Korea; 3 Institute for Biomedical Convergence, Catholic Kwandong University International St. Mary’s Hospital, Incheon, South Korea; 4 Department of Endocrinology and Metabolism, Kangbuk Samsung Hospital, Sungkyunkwan University School of Medicine, Seoul, South Korea; 5 Department of Medicine, University of Leipzig, Leipzig, Germany; 6 Osteoneurogen, Inc. #705 Ace High-end Tower 9th 233, Gasandigital-1-ro, Geumcheon-gu, Seoul 08501 South Korea

## Abstract

Elevated circulating Retinol-binding protein 4 (RBP4) has been associated with insulin resistance, dyslipidemia, and hypertension. However, many commonly used RBP4 ELISAs have limited dynamic range. We therefore developed an enzyme-linked immunosorbent sandwich assay (ELISA) employing a novel immunoglobulin A (IgA)-type capture mAb called AG102 instead of IgG subtypes, which was selected for its stability, capture efficiency, and specificity for human RBP 4. These features of RBP4 have hampered the development of quantitative immunological assays. Molecular analysis of AG102 revealed IgA heavy and light chains and a J chain, as expected. AG102 demonstrated notable detection of both bacterial- and HEK293-expressed RBP4 in Western blots. Serial and internal deletion experiments suggested that a putative epitope may be located in the first 35 amino acids of the mature RBP4. Compared with commercial ELISAs, the AG102-based system exhibited more significant recovery of RBP4 from serum or urine at any given dilution factor. To substantiate its quantitation capacity, comparison between RBP4 measurements from quantitative western blots and the AG102-based ELISA demonstrated a significant correlation (R^2^ = 0.859). After measurement for those analytes, our data suggested that IgA-based ELISA could be adapted for quantitative measurement of those analytes existing as major serum proteins or as multi-protein complexes like RBP4.

## Introduction

Insulin resistance contributes to the progression from normal glucose tolerance (NGT) to impaired fasting glucose (IFG), impaired glucose tolerance (IGT), and type 2 diabetes mellitus (T2D)^1^. Serum retinol binding protein (RBP4) is increased in insulin-resistant states and highly associated with both the magnitude of insulin resistance and individual components of the metabolic syndrome and the risk to develop coronary heart disease in humans^2–5^. Increased serum RBP4 causes insulin resistance in mice by interfering with insulin signaling in skeletal muscle and the liver^6^. In addition, RBP4 activates antigen-presenting cells in adipose tissue, thereby eliciting low-grade inflammation and leading to systemic insulin resistance^7^. This suggests that RBP4 is an important regulator for immunometabolism. RBP4 is expressed in the liver and adipose tissue, and adipocyte *RBP4* mRNA is strongly increased in human obesity, particularly within visceral fat deposits^5^. Recent studies indicate that a functional polymorphism of the *RBP4* promoter causes increased adipocyte RBP4 expression and is associated with increased serum RBP4 concentrations and risk for type 2 diabetes^8–10^. Noteworthy, some studies did not find a relationship between circulating RBP4 level and insulin resistance^11–13^. These discrepant findings may reflect differences in study subjects, differences in methods used for quantifying insulin resistance, or technical problems inherent in the current methods used for measuring RBP4^11,14^.

Accordingly, quantitative Western (q-western) blotting has remained a preferred method for RBP4 serum analyses^14^. Several factors may influence the ability of different assays to accurately quantify RBP4. In healthy individuals, the majority of circulating RBP4 exists in a stable 1:1 complex with Transthyretin (TTR), also known as prealbumin, a 56 kDa plasma protein^15^. RBP4 binds TTR with high affinity via sites on both proteins that have been identified by X-ray crystallography of the complex^15^. RBP4 ·TTR binding stabilizes RBP4 in circulation by preventing its glomerular filtration^16^. The carboxyl terminus of RBP4 forms part of the TTR binding interface, and carboxyl terminus-proteolyzed RBP4 variants display reduced affinity for TTR^15^. Proteolyzed RBP4 appears to undergo rapid renal clearance, since it is abundant in urine, but difficult to detect in normal individuals. Conversely, proteolyzed RBP4 variants have been found to accumulate in the serum of individuals with end-stage renal disease who lack an effective clearance mechanism^14,17,18^. It is likely that the relative concentrations of RBP4 and TTR in circulation, the affinity of RBP4·TTR binding, and the variable presence of proteolyzed RBP4 can influence the ability of immunoassays to accurately quantify RBP4. In addition to this diagnostic pitfall, RBP4 serum concentration is typically higher than a few hundred µg/ml, resulting in the so-called “hook effect” even after significant dilution^19^.

Here, we report the development of a new enzyme-linked immunosorbent sandwich assay (ELISA) using a novel IgA mAb for efficient capture of human RBP4 from serum or urine. Due to its dimeric structure, IgA’s antigen-capture ability is superior to that of IgG. The current IgA mAb was selected for its reactivity with a specific epitope of RBP4 at the N-terminus, which is not located within the RBP4·TTR binding interface^15^. The subsequent extensive validation assays exhibited greater dynamic recovery of RBP4 from serum and urine than other commercial assays. The distribution features of serum recovery via this IgA-based ELISA were comparable to those of q-Western blotting. We propose that this novel IgA-based immunoassay should be used to define the relationships between urinary RBP4 level and clinical measures of insulin-glucose homeostasis and urinary albumin excretion.

## Methods

### Production of recombinant RBP4 proteins and epitope mapping

A cDNA sequence encoding the mature peptide of human RBP4 was amplified with the primer pairs shown in Table 1, and a FLAG tag was incorporated at the N_-_ terminus of the peptide. The augmented cDNA of RBP4 was then cloned into pAGNF and pET-21a(+) vectors (Novagen, Madison, WI) and *Nde* I/*Xho* I were used for constructing the expression vector. pAGNF is driven by the CMV early promoter, and protein secretion is facilitated by the plasminogen activator inhibitor type I (PAI-1) leader peptide^20^. FLAG-RBP4 was expressed in a human embryonic kidney cell line, HEK-293, and purified from conditioned media using an anti-FLAG column (Sigma-Aldrich, St Louis, MO, USA). 6 × His-tagged RBP4 was purified via a Ni Sepharose column. A polyclonal antibody (pAb) and mAbs were produced by immunizing rabbits and BALB/c mice, respectively, with recombinant 6X His-tagged human RBP4, and hybridomas were generated and cloned according to standard protocols^21^. A panel of mAbs produced by different clones was screened with respect to reactivity to FLAG-RBP4. For epitope mapping, two deletion versions of recombinant human RBP4 lacking amino acid residues 1–35 or 138–183 and an internal deletion mutant lacking amino acid residues 36–78 were created by PCR with appropriate primer sets (Supplementary Table II) and subcloned into pET-21a(+). Protein expression was confirmed by Western blotting with AG102 (AdipoGen Life Sciences, San Diego, CA). For these studies, residue 1 refers to the first amino acid following the cleaved 18-amino acid signal peptide of pro-RBP4.

**Table 1:** Isotyping of anti-human RBP4 mAb AG102 by ELISA.

Isotype	IgG1	IgG2a	IgG2b	IgG3	IgA	IgM	Kappa	Lambda
Absorbance	0.119	0.102	0.094	0.186	**1**.**982**	0.256	**1**.**546**	0.116

To confirm the isotypes comprising AG102, isotyping ELISAs were performed, rendering IgA as a heavy chain and Kappa as a light chain.

### Molecular cloning and sequence analysis of the cDNA encoding AG102

The cDNAs for the variable genes of the heavy and light chains of AG102 (AdipoGen Life Sciences, San Diego, CA) were amplified using the 5’ rapid amplification of cDNA ends (5’RACE) method. Primer sequences are presented in Supplementary Table 1. Briefly, total RNA was isolated from AG102 monoclonal antibody-expressing hybridomas using TRIzol reagent (Life Technologies, Carlsbad, CA, USA). The 5′ RACE System (SMARTer^TM^ RACE cDNA Amplification Kit, Clontech, Mountain View, CA, USA) was used according to the manufacturer’s instructions.

### Development of a quantitative human RBP4 ELISA and measurement of RBP4 concentrations in serum and urine

A sandwich ELISA format was designed using AG102 as the capture antibody and pAb (AdipoGen Life Sciences, San Diego, CA) for detection, with FLAG-tagged RBP4 as a standard. The wells of a microtiter plate were first coated with 5 μg/ml AG102 at 37 °C for 1 h. Next, 100 μl of human serum (diluted 1:50,000), human urine (diluted 1:100), or diluted standard was applied to the AG102-coated wells and incubated at 37 °C for 1 h. The wells were then washed three times with 0.05% Tween-20 in phosphate-buffered saline (PBST). Next, 100 µl of the purified pAb (5 μg/ml) was added per well and incubated at 37 °C for 1 h, and the wells were washed three times with PBST. Detection was performed by incubating each well with 100 µl of horseradish peroxidase (HRP)-conjugated streptavidin (Zymed) diluted 1:1,000 in PBS at 37 °C for 1 h, washing three times in PBS, and conducting a colorimetric reaction for 20 min with the HRP substrate 2,2′-azino-bis(2-ethylbenzothiazoline-6-sulfonic acid) (Pierce, Rockford, IL). Optical density was measured at the product’s maximal absorbance of 450 nm. All participants provided written informed consent. The Institutional Review Board of Kangbuk Samsung Hospital in Korea approved the study protocol that was in accordance with the Declaration of Helsinki.

### Quantitative Western blots

Predetermined recombinant RBP4 proteins expressed by HEK293 cells were resolved on a polyacrylamide gel in parallel with five serum samples whose concentrations were determined by the IgA-based ELISA. After probing the Western blot filters with a rabbit anti-RBP4, phospho-image analysis was conducted using an image analyzer (LAS-1000Plus, FUJIFILM, Japan). Quantitation is represented in AU/mm^2^ according to the manufacturer’s instructions.

## Results

### Isolation of an IgA mAb specifically recognizing human serum RBP4

**Figure 1:**
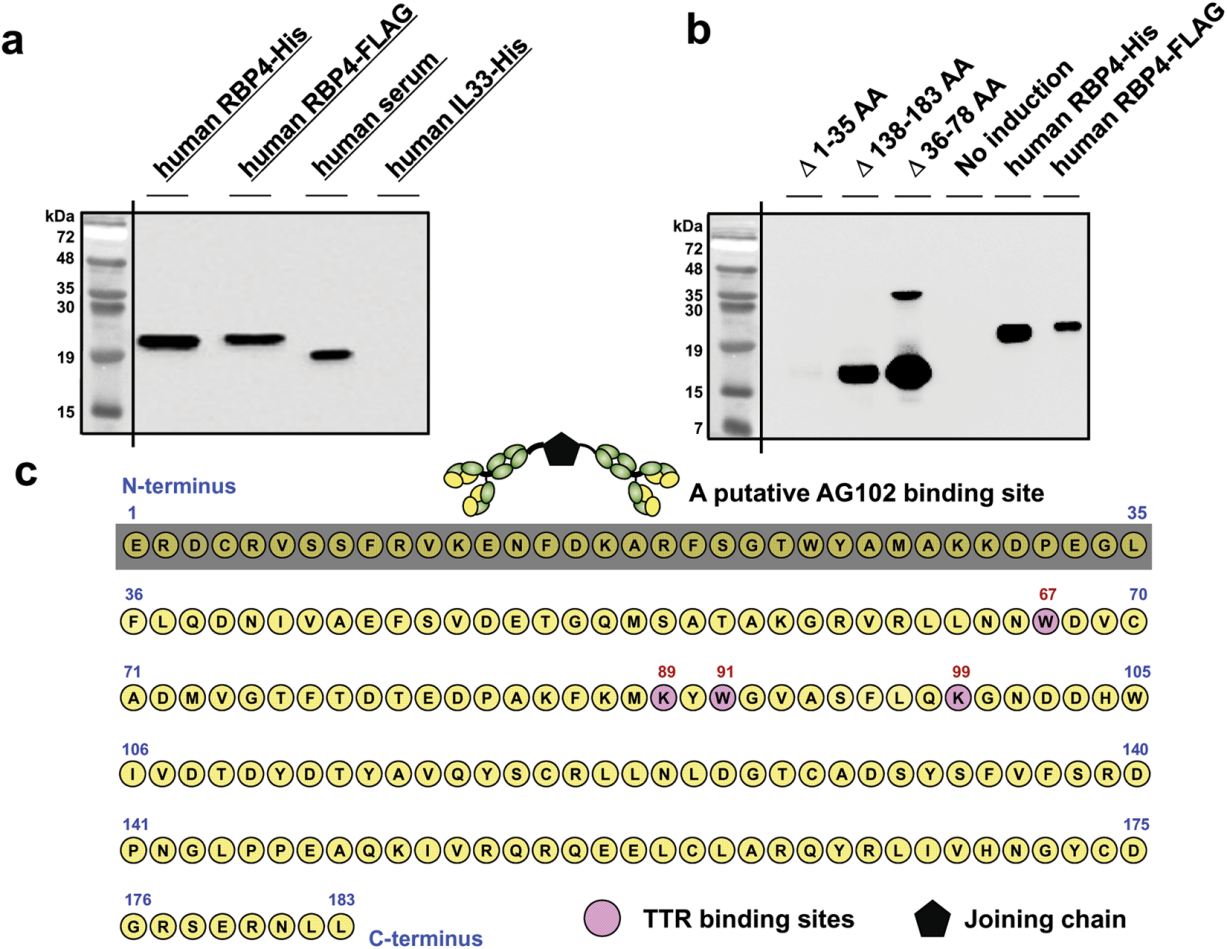
Development of a quantitative ELISA measuring RBP4 concentration in urine using a novel mAb. (**a**) The reactivity of the capture mAb AG102 was tested using Western blot with both recombinant and serum RBP4 proteins along with a control recombinant protein. (**b**) After making a serial deletion mutant RBP4 gene, these expression constructs were introduced in BL21, an IPTG-inducible *E*.*coli* host. Deletion points are indicated in Supplementary Table III, resulting in three deleted recombinant RBP4s. Upon IPTG induction, Western blot was conducted along with the use of a human serum or FLAG-RBP4. As a negative control, non-induced E.coli cell lysates were also used for the specificity of the Western blot. (**c**) The amino acids of human RBP4 are represented. Epitopes within the first 35 amino acid residues (bracketed) are recognized by AG102, which comprise four antigen binding sites and a joining region (J). The four critical amino acid residues involved in the TTR binding are depicted by red circles.

**Figure 2:**
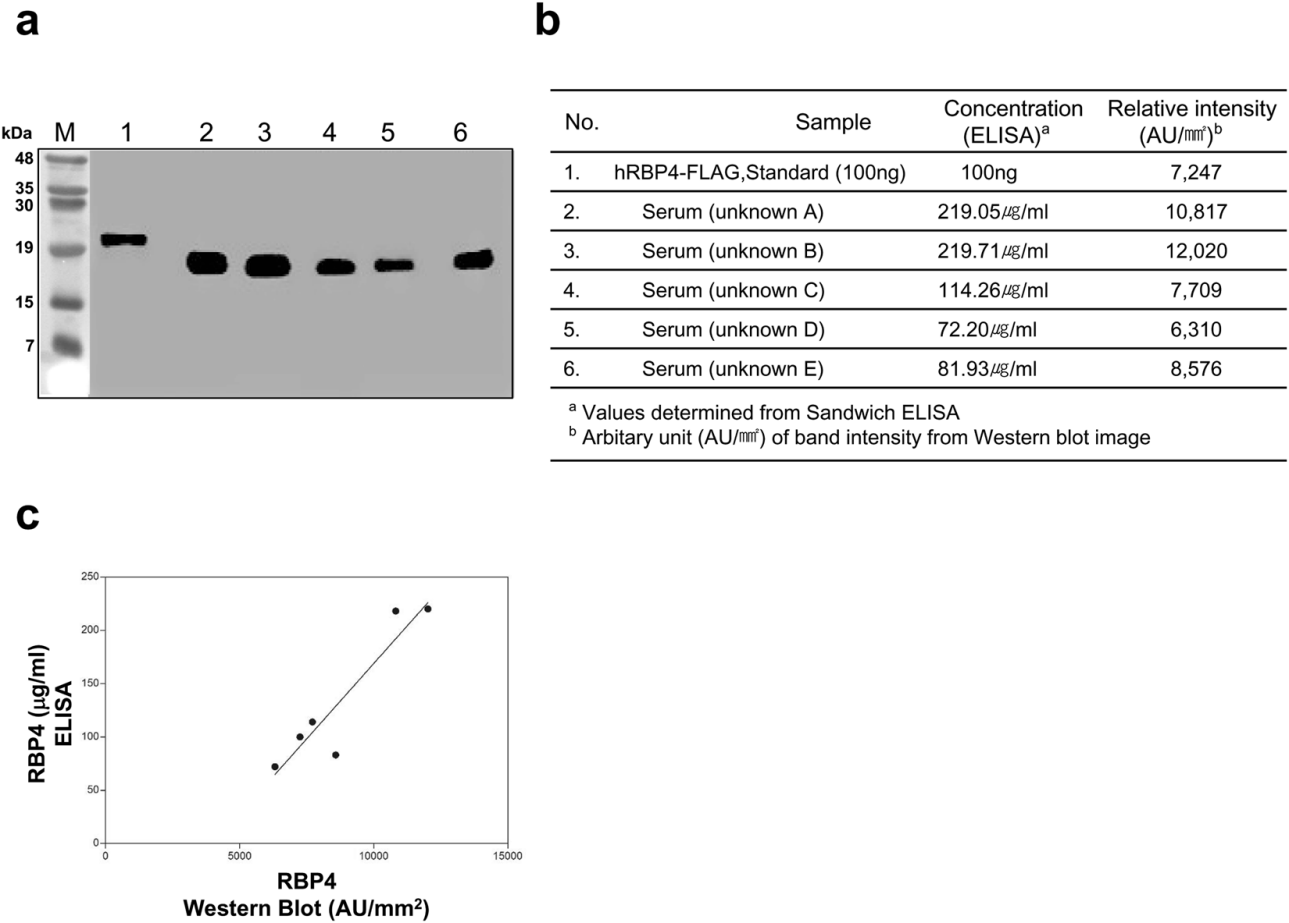
Correlation of the IgA-based ELISA with q-Western blotting. (**a**) A control sample representing 100 ng of recombinant RBP4 (lane 1) and five human serum samples (1 μl each, lanes 2 through 6) were subjected to Western blotting using AG102 as a detector antibody. (**b**) Five serum samples from normal donors and the recombinant RBP4 protein were tested simultaneously with the standard, and the band intensity values from Western blot images of (**a**) were interpolated to determine RBP4 quantification. Using a phosphor-image analyser, the reactive area (denoted by AU/mm^2^) was calculated and plotted with the RBP4 concentrations determined by the IgA-based ELISA. (**c**) Quantification results for both methods. Coefficient of determination (R^2^) is indicated.

As shown in Fig. 1a, the mAb AG102 readily detected serum RBP4 and two differently-tagged forms of recombinant RBP4 by Western blotting. Consistent with epitope mapping, AG102 did not recognize an N-terminus RBP4 truncation mutant (Δ1–35 AA) in Western blotting, but did recognize the internal deletion and C-terminal deletion mutant RBP4 proteins with normal avidity (Δ36–78 AA and Δ 138–183 AA, respectively; Fig. 1b). The AG102 isotype was determined to be IgA associated with a Kappa light chain as shown in Table 1 and was further confirmed with 5’ RACE PCR cloning and sequencing of the variable domains and leader sequences. Each variable region of the heavy or light chain contained well-conserved complementarity-determining regions (CDRs) (Supplementary Figure 1 and Fig. 2). Several monoclonal anti-RBP4 antibodies were generated and screened for FLAG-RBP4 immunoreactivity by Western blotting and secondarily mapped to specific domains of RBP4. An optimal clone, identified as AG102, produced a mAb mapping to an epitope near the N-terminus of RBP4 within amino acids 1–35 (Fig. 1c), which is a region located on the surface of RBP4 and not involved in RBP4·TTR binding^15^. The cDNA sequence corresponding to the J chain was also verified. These findings confirm that AG102 is a monoclonal IgA that recognizes an N-terminus epitope of RBP4 that is likely to be exposed on the surface of RBP4 and unlikely to be affected by either high-affinity RBP4·TTR interactions or RBP4 C-terminal proteolysis, factors proposed to contribute to variability and technical limitations in other assays^14,15^.

### Development of a novel IgA-based ELISA

**Figure 3:**
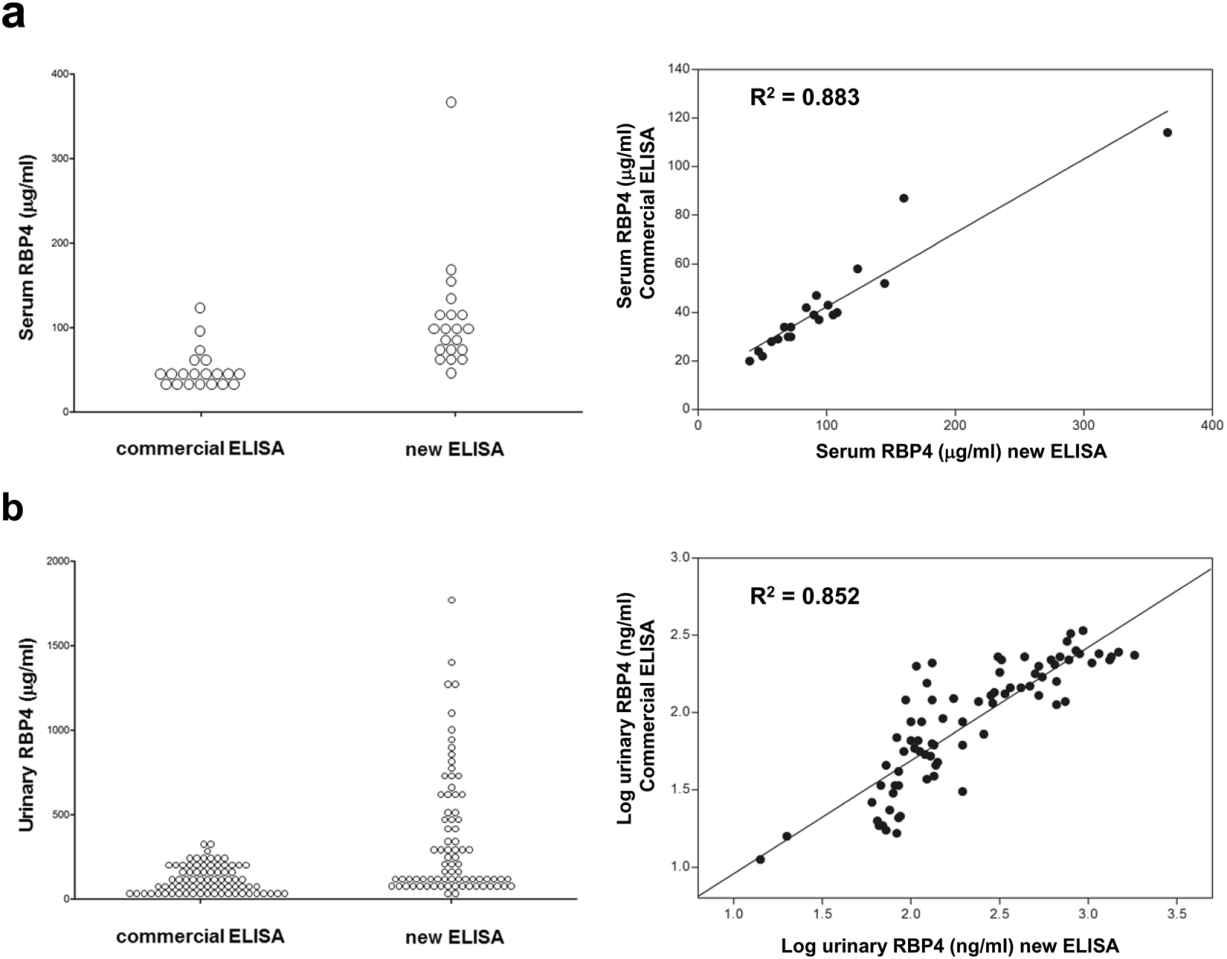
Comparison of correlation and dynamic serum or urine recovery associated with the IgA-based ELISA with commercial ELISAs. (**a**) Twenty sera were selected and subjected to the IgA-based and commercial RBP4 ELISAs. Correlation and dynamic serum recovery are shown. (**b**) Urine samples from 78 volunteers were collected and subjected to ELISA as described above. Correlation and dynamic urine recovery are shown.

A classic sandwich ELISA using AG102 as the capture antibody was created and used to measure RBP4 in five selected human sera exhibiting a broad range of RBP4 concentrations. The maximum sensitivity was 380 pg/ml. Intra-assay C.V. was 1.7–3.7% (Supplementary Table III), and inter-assay C.V. was 7.0–8.8%. (Supplementary Table IV). Spike recovery was 95–115%, and linearity was 90–100% over the chosen dilution range. As shown in Fig. 2a,b, RBP4 measured by ELISA correlated well with RBP4 measured by q-Western blotting (R^2^ = 0.859). To compare the correlation and dynamic serum recovery associated with the IgA-based ELISA, 20 serum samples were subjected to quantification with the IgA ELISA and with commercial ELISAs. As seen in Fig. 3a, the two systems were significantly correlated (R^2^ = 0.883, *P* < 0.001), indicating that the novel assay was at least comparable in performance to commercial assays with regard to measurement of serum RBP4 concentration^14^. However, the dynamic range of serum RBP4 concentrations detectable by the IgA-based ELISA was significantly larger than that of the commercially-available ELISAs. We routinely observed that the degree of serum recovery via the IgA-based ELISA was 1.5- to 2.0-fold larger than that of the commercial assays.

Urinary RBP4 concentrations in 78 subjects were measured in parallel using the new (IgA-based) ELISA and two commercial assays (Immnodiagnostik, Bensheim, Germany; R&D systems, Minneapolis, MN, USA). Measurements of urinary RBP4 with the new ELISA correlated significantly with the commercial assay (R^2^ = 0.852, *P* < 0.001, Fig. 3b). However, the IgA-based ELISA displayed a greater median and wider range of values than the commercial assays (new ELISA - median 163.96 ng/ml, range 93.87–537.03 ng/ml; commercial assay - median 112.41 ng/ml, range 43.40–206.53 ng/ml; *P < 0*.*05*). In addition to this unique feature, intra- and inter-assay coefficients of variation were below 4% and 8%, respectively, suggesting that this IgA-based ELISA was highly stable (Supplementary Tables III and IV). Prior work comparing commercial assay performance for serum RBP4 measurements relative to the gold standard q-Western blotting method revealed that several assays tended to underestimate serum RBP4 concentration, especially in insulin-resistant subjects^14^. This might result from specific assay characteristics and/or intrinsic characteristics of RBP4 present in insulin resistance such as altered RBP4·TTR interactions or C-terminal RBP4 proteolysis^15,16^. The greater dynamic range exhibited by the new ELISA may reflect the superior characteristics of the AG102 IgA monoclonal antibody used for capture.

## Discussion

Here, we demonstrated a newly developed IgA mAb-based ELISA, which is a semi-quantitative tool for measuring the major serum protein, RBP4, upon a proper single dilution. To our knowledge, this new immunologic assay is the most convenient option that has been described to replace q-Western blot or LC/MS-MS^22^. As RBP4 is a novel biomarker for assessing the severity of whole-body insulin resistance and associated immune-metabolic abnormalities, this robust and quantitative assay would be a useful diagnostic tool. Structural studies of the RBP4·TTR complex indicate that, *in vitro*, up to two RBP4 molecules may form a molecular complex with tetrameric TTR via a docking site comprising four critical RBP4 amino acid residues (Trp^67^, Lys^89^, Trp^91^, and Lys^99^)^16^. In addition, other studies^15^^,^^23^ have demonstrated that the RBP4 C-terminus, which plays an important role in forming the RBP4·TTR interface, is proteolyzed to varying degrees in serum and urine. In addition to influencing the RBP4·TTR interaction, proteolysis of RBP4 may influence RBP4 conformation. Together, these factors could affect the accessibility of any given monoclonal antibody or affinity-purified polyclonal antibody to native RBP4 obtained from serum or urine, especially when studied under diluted conditions, resulting in variable recoveries of RBP4.

Our analysis clearly shows that the IgA mAb AG102, which is directed toward the extreme N-terminus of RBP4 far from the regions of RBP4 known to be involved in TTR binding, displays a superior recovery of urinary RBP4 compared to commercial RBP4 assays utilizing IgG antibodies. In addition, it is likely that the greater tolerance of IgA for a wide range of pH values and the greater number of antigen binding sites on IgA may provide a superior capture antibody for measuring urinary RBP4. Thus, this newly developed IgA-based ELISA will provide a more reliable method for measuring urinary RBP4 and thereby enhance our understanding of the relationship between RBP4 and impaired insulin-glucose homeostasis. Since our cDNA sequences revealed the CDR and framework regions, these regions can be replaced by a specific antigenic-binding site and engineered to generate synthetic IgA-based mAbs, thereby helping improve this IgA technology.

## Electronic supplementary material

Supplementary information
