# Supplementary material for: Development of a mouse IgA monoclonal antibody-based enzyme-linked immunosorbent sandwich assay for the analyses of RBP4
Source: Sci Rep. 2018 Feb 7;8:2578. doi: 10.1038/s41598-018-20762-x (PMC5803225; doi:10.1038/s41598-018-20762-x)
Supplement: Supplementary file 1 — Supplementary information [file 41598_2018_20762_MOESM1_ESM.doc]

**Development of a mouse IgA monoclonal antibody-based enzyme-linked immunosorbent sandwich assay for the analyses of RBP4**

Nam Seok Lee^1†^, Han Soo Kim^2,3†^, Se Eun Park^4^, Matthias Blüher^5^, Cheol-Young Park^4*^, Byung-Soo Youn^6*^

^1^Green Cross Medical Science, Umseong-gun, Chungcheongbuk-do, South Korea.

^2^Department of Biomedical Sciences, Catholic Kwandong University College of Medicine, Gangneung-si, Gangwon-do, South Korea.

^3^Institute for Biomedical Convergence, Catholic Kwandong University International St. Mary’s Hospital, Incheon, South Korea.

^4^Department of Endocrinology and Metabolism, Kangbuk Samsung Hospital, Sungkyunkwan University School of Medicine, Seoul, South Korea.

^5^Department of Medicine, University of Leipzig, Leipzig, Germany

^6^Osteoneurogen, South Korea.

^†^NS Lee and HS Kim contributed equally to this study.

**^*^Corresponding Author:**

Byung-Soo Youn

Osteoneurogen, Inc. #705 Ace High-end Tower 9th 233, Gasandigital-1-ro, Geumcheon-gu, Seoul 08501, South Korea

Telephone: 82-2-6267-2737; Fax: 82-2-6267-2740

E-mail: byung4jc@gmail.com

and

Cheol-Young Park

Division of Endocrinology and Metabolism, Department of Internal Medicine, KangbukSamsung Hospital, Sungkyunkwan University School of Medicine, 29 Saemunan-ro, , Jongno-gu, Seoul 03181, South Korea

Telephone: 82-2-2001-1869; Fax: 82-2-2001-1588

E-mail: [cydoctor@chol.com](mailto:cydoctor@chol.com)

http://orcid.org/0000-0002-9415-9965

*Supplementary Tables*

**Table I. RACE PCR primers to isolate a full-length cDNA encoding AG102**

|  |  | Primer sequence |
| --- | --- | --- |
|  |  |  |
|  | Universal primer (UP) | Forward 5'-CTA ATA CGA CTC ACT ATA GGG C-3' |
|  | VH Gene specific primer (GSP) | Reverse 5'-CTC AGG ATT TCT CAG GCC ATT CAG-3' |
|  | VL Gene specific primer (GSP) | Reverse 5'-GCT GTC TTT GCT GTC CTG ATC AGT-3' |
|  |  |  |

The primers, gene-specific primers (GSP1 and GSP2), encoding VH and VL, respectively, were designed by retrieving mouse immunoglobulin A sequences in the Gene bank and their sequences are shown in Table II. 5’ RACE amplified products were cloned into the T&A cloning vector (T&A cloning vector kit, RBC Biosciences Corp., New Taipei City, Taiwan) and sequenced. The hybridoma producing AG102 was deposited to the Korean Collection for Type Cultures (KCTC) under a deposit permission number, KCTC 11630BP, and its cDNA sequences were described in the patent with registration number 10-1042579. Immunoglobulin fractions were prepared from serum and ascites.

2

**Table II.** **Primer sequences**

|  | **Primer Sequence** |
| --- | --- |
|  |  |
|  | Forward 5’-GGGAATTCCATATGTTTCTGCAGGACAACATCGTC-3’ |
| **Δ1-35 AA** | Reverse 5’-CCCGCTCGAGCTAGTGGTGGTGGTGGTGGTGCAAAAGGTTTCTTTCTGATCTGCC-3’ |
|  |  |
|  | Forward 5’-GGGAATTCCATATGGAGCGCGACTGCCGAGTGAGC-3’ |
| **Δ36-78 AA** | Reverse 5’-GAGGCCCTCGGGGTCCTTCTT-3’ |
|  |  |
|  | Forward 5’-GACACCGAGGACCCTGCCAAG-3’ |
|  | Reverse 5’-CCCGCTCGAGCTAGTGGTGGTGGTGGTGGTGCAAAAGGTTTCTTTCTGATCTGCC-3’ |
|  |  |
|  | Forward 5’-GGGAATTCCATATGGAGCGCGACTGCCGAGTGAGC-3’ |
| **Δ138-183 AA** | Reverse 5’-CCCGCTCGAGCTAGTGGTGGTGGTGGTGGTGAAACACGAAGGAGTAGCTGTC-3’ |
|  |  |

To determine a potential epitope recognized by AG102 three kinds of RBP4 deletion mutants

were generated via various PCR primer sets

**Table III.** **Intra-Assay (precision within an assay)**

| **Sample** | **Mean (µg/ml)** | **SD (µg/ml)** | **CV (%)** |
| --- | --- | --- | --- |
| 1 | 169.0 | 6.3 | 3.7 |
| 2 | 178.5 | 3.1 | 1.7 |
| 3 | 127.4 | 2.4 | 1.9 |
| 4 | 141.8 | 3.5 | 2.5 |
| 5 | 66.8 | 2.3 | 3.4 |
| 6 | 77.7 | 2.8 | 3.6 |

Using six human serum samples, intra-assay variation assessment was performed eight times to calculate standard deviation (SD) and coefficient of variance (CV) values.

**Table IV.** **Inter assay (precision between assay)**

| **Sample** | **Mean (µg/ml)** | **SD (µg/ml)** | **CV (%)** |
| --- | --- | --- | --- |
| 1 | 194.7 | 14.4 | 7.4 |
| 2 | 192.4 | 15.5 | 8.1 |
| 3 | 141.2 | 11.6 | 8.2 |
| 4 | 154.5 | 9.2 | 5.9 |
| 5 | 89.8 | 7.9 | 8.8 |
| 6 | 73.3 | 5.2 | 7.1 |

3

Using six human serum samples, inter-assay variation assessment was performed eight times to calculate standard deviation (SD) and coefficient of variance (CV) values.

**Table V.** **Human RBP4 ELISA specificity**

| **Analyte** | **Max. Conc.**  **(ng/ml)** | **ELISA**  **(OD)** | **Cross**  **Reactivity (%)** |
| --- | --- | --- | --- |
| Human RBP4 | 10 | 1.04 | 100 |
| Mouse RBP4 | 100 | 0.06 | N. R. |
| Rat RBP4 | 100 | 0.06 | N. R. |
| Human adiponectin | 100 | 0.05 | N. R. |
| Human Resistin | 100 | 0.06 | N. R. |
| Human Vaspin | 100 | 0.06 | N. R. |
| Human Clusterin | 100 | 0.06 | N. R. |
| Human Leptin | 100 | 0.05 | N. R. |
| Human IL-33 | 100 | 0.05 | N. R. |
| Human GPX3 | 100 | 0.02 | N. R. |
| Human Progranulin | 100 | 0.03 | N. R. |
| Human FABP4 | 100 | 0.03 | N. R. |
| Human ANGPTL3 | 100 | 0.04 | N. R. |
| Human ANGPTL4 | 100 | 0.04 | N. R. |
| Human ANG1 | 100 | 0.05 | N. R. |
| Human ANG2 | 100 | 0.03 | N. R. |
| Human Visfatin | 100 | 0.03 | N. R. |
| Mouse Visfatin | 100 | 0.01 | N. R. |

**N. R.: No Cross-Reactivity**

Human RBP4 ELISA specificity. No cross-reactivity was noted when mouse and rat sera were tested. Seventeen recombinant proteins including both human and mouse adipokines or cytokines at concentrations of 100 ng/ml were examined for their cross-reactivity. N. R. stands for no cross-reactivity. Specificity was tested by assessing reactivity with mouse RBP4 and rat RBP4, and a variety of unrelated purified analytes, including: human adiponectin,

4

visfatin, IL-33, leptin, vaspin, fatty acid binding protein 4 (FABP4), angiopoietin-like protein 3 (ANGPTL3), angiopoietin-like protein 4 (ANGPTL4), angiopoietin 1, angiopoietin 2, glutathione peroxidase 3 (GPX3), clusterin, resistin, progranulin, and mouse visfatin (Supplementary Table III). We compared the newly established ELISA assay with two commercially available sandwich ELISA assays; Immunediagnostik (Bensheim, Germany) and R&D Systems (Minneapolis, MN) using either serum or urine samples according to the supplied manuals. Serum TTR was measured by a commercial ELISA assay (Immunediagnostik). All participants provided written informed consent. This study was approved by the Institutional Review Board of Kangbuk Samsung Hospital. The current IgA-based RBP4 ELISA is commercially available (AdipoGen Life Sciences / AG-45A-0035Y).

5

*Supplementary Figures*


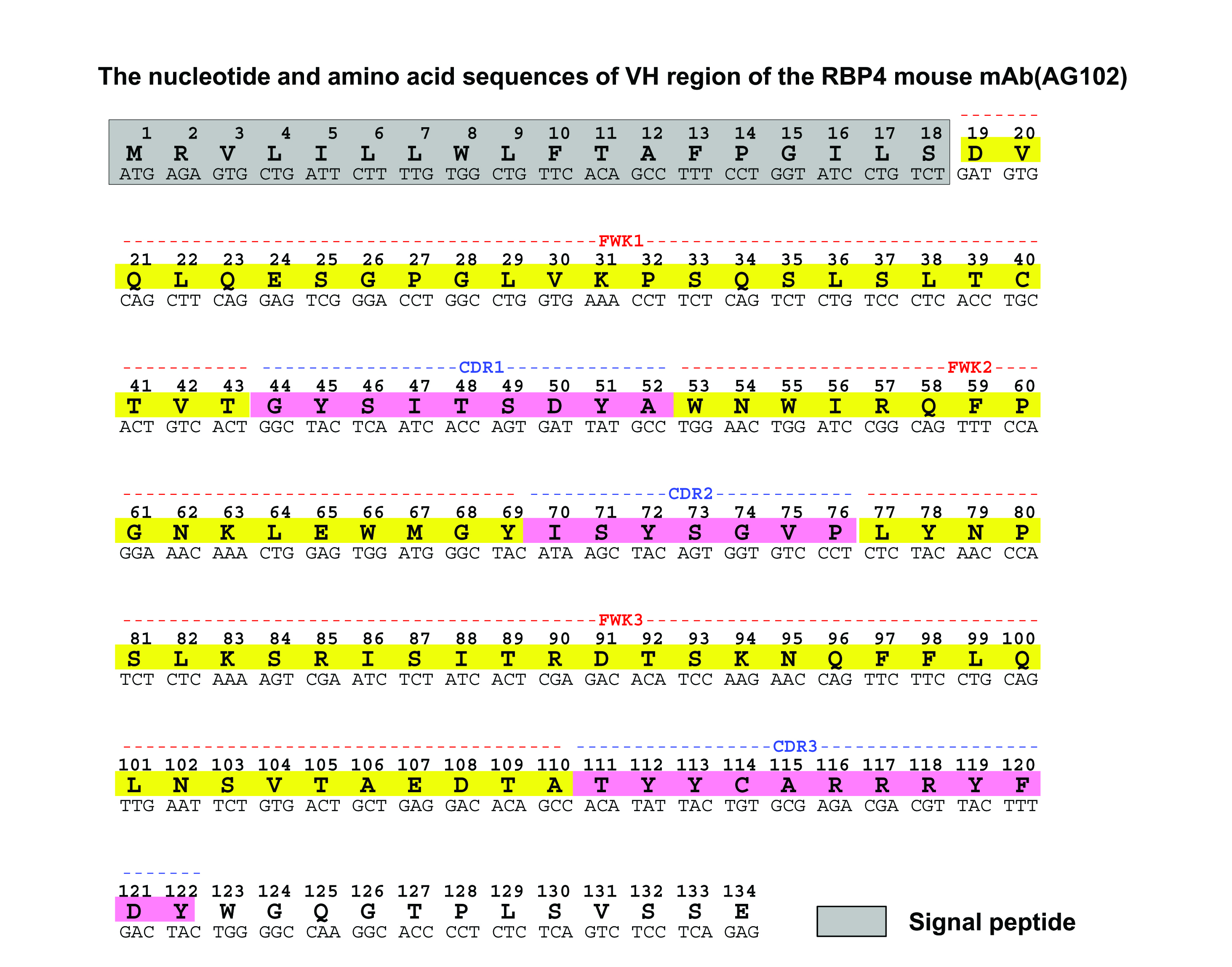


**Figure 1. The nucleotide and amino acid sequences of VH region of the RBP4 mouse mAb**

**(AG102)** Amino acids were deduced from the entire cDNA sequence encoding variable region of the heavy chain of AG102. A putative signal peptide was represented by a grey box. Framework regions were denoted by yellow whereas CDRs were expressed by pink.

6


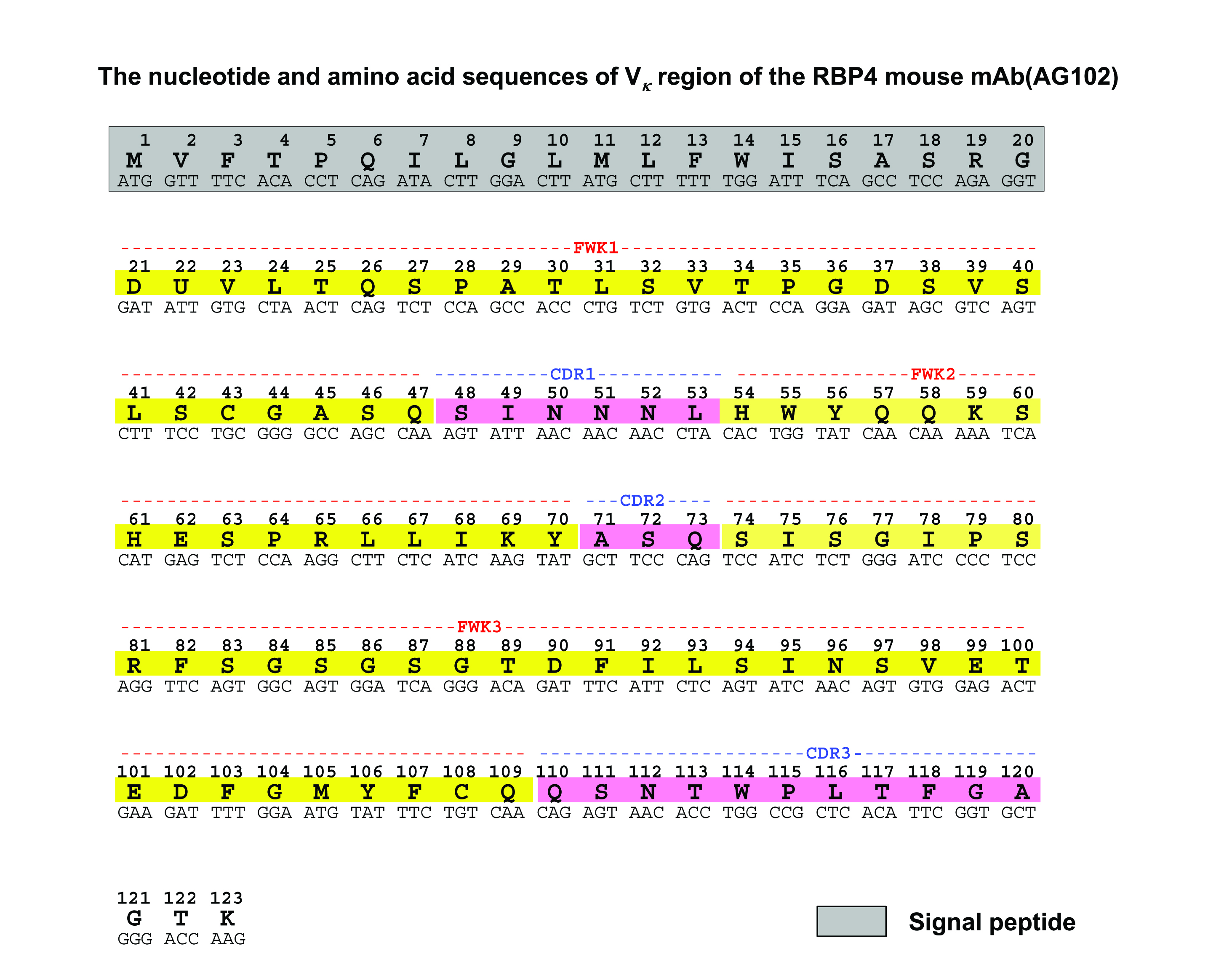


**Figure 2. The nucleotide and amino acid sequences of V*_κ_* region of the RBP4 mouse mAb**

**(AG102)** Amino acids were deduced from the entire cDNA sequence encoding variable region of the kappa light chain of AG102. A putative signal peptide was represented by a grey box. Framework regions were denoted by yellow whereas CDRs were expressed by pink.

7
